# Association between pregnancy timing and disability outcomes in women with mild relapse-onset multiple sclerosis

**DOI:** 10.1177/13524585261464055

**Published:** 2026-07-29

**Authors:** Jessica Shipley, Chao Zhu, Paul G Sanfilippo, Olga Skibina, Tomas Kalincik, Raed Alroughani, Bruce V Taylor, Richard Macdonell, Vicki E Maltby, Jeannette Lechner-Scott, Heidi N Beadnall, Anneke van der Walt, Helmut Butzkueven, Vilija G Jokubaitis

**Affiliations:** Department of Neuroscience, School of Translational Medicine, Monash University, Melbourne, VIC, Australia; Department of Neurology, Alfred Health, Melbourne, VIC, Australia; Department of Neuroscience, School of Translational Medicine, Monash University, Melbourne, VIC, Australia; Department of Neuroscience, School of Translational Medicine, Monash University, Melbourne, VIC, Australia; Department of Neurology, Alfred Health, Melbourne, VIC, Australia; Department of Neurology, Box Hill Hospital, Box Hill, VIC, Australia; CORe, Department of Medicine, The University of Melbourne, Melbourne, VIC, Australia; Neuroimmunology Centre, Department of Neurology, The Royal Melbourne Hospital, Melbourne, VIC, Australia; Department of Neurology, Al-Amiri Hospital, Kuwait City, Kuwait; Multiple Sclerosis – Menzies Institute for Medical Research, University of Tasmania, Dynnyrne, TAS, Australia; Department of Neurology, Austin Health, Heidelberg, VIC, Australia; Department of Neurology, John Hunter Hospital, New Lambton Heights, NSW, Australia; Hunter Medical Research Institute, School of Medicine and Public Health, The University of Newcastle, New Lambton Heights, NSW, Australia; Department of Neurology, John Hunter Hospital, New Lambton Heights, NSW, Australia; Hunter Medical Research Institute, School of Medicine and Public Health, The University of Newcastle, New Lambton Heights, NSW, Australia; Multiple Sclerosis Clinic, Brain and Mind Centre, The University of Sydney, Camperdown, NSW, Australia; Department of Neuroscience, School of Translational Medicine, Monash University, Melbourne, VIC, Australia; Department of Neurology, Alfred Health, Melbourne, VIC, Australia; Department of Neuroscience, School of Translational Medicine, Monash University, Melbourne, VIC, Australia; Department of Neurology, Alfred Health, Melbourne, VIC, Australia; Department of Neuroscience, School of Translational Medicine, Monash University, Melbourne, VIC, Australia; Department of Neurology, Alfred Health, Melbourne, VIC, Australia

**Keywords:** Multiple sclerosis, pregnancy, disability, outcomes research

## Abstract

**Background:**

Pregnancy does not adversely affect disability outcomes in women with multiple sclerosis (MS). However, it remains uncertain whether pregnancy is associated with slower disability accumulation, and whether this association differs according to the timing of pregnancy during the disease course.

**Methods:**

This observational cohort study used prospectively ascertained data in the international MSBase Registry (1991–2022), supplemented with retrospective surveys detailing participants’ complete pregnancy histories. The study included women with mild relapse-onset MS, defined as an Expanded Disability Status Scale (EDSS) score of 2.5 or below. A flexible parametric survival model was used to assess the time-varying effect of live-birth pregnancy on reaching sustained EDSS 3.

**Results:**

We included 2079 women, of whom 571 had a live-birth pregnancy after baseline. Live-birth pregnancies occurring within the first 4.42 years of follow-up were associated with longer time to sustained EDSS 3. At 10 years, live birth was associated with an absolute cumulative difference of 0.51 years in time to this disability milestone (95% confidence interval (CI): 0.23, 0.79). Among women with live births, postpartum relapses were associated with shorter time to sustained EDSS 3 (hazard ratio (HR): 1.61; 95% CI: 1.02, 2.55).

**Conclusion:**

Live-birth pregnancy occurring earlier in follow-up was associated with a longer time to disability accrual.

## Introduction

Multiple sclerosis (MS) is an autoimmune, neurodegenerative condition with a female overrepresentation of approximately three females to every one affected male. It is often diagnosed between the ages of 20 and 40, coinciding with key reproductive years, and therefore, family planning discussions are an essential part of MS care. Over recent years, the management of MS pregnancy through planning, pregnancy and postpartum has significantly advanced due to better understanding of disease activity,^1,2^ and increased evidence for the use of specific disease-modifying therapies (DMTs) during these periods.^1,3,4^ However, the long-term impact of pregnancy on the MS disease course remains unclear, adding complexity to family planning discussions.^5^

Tolerogenic pregnancy-mediated immune adaptations^6^ are associated with reductions in MS relapse activity during pregnancy.^7–11^ However, factors such as resolution of this immune tolerance and falls in pregnancy-related hormones after delivery are associated with an increased relapse risk postpartum. While the proportion of women experiencing postpartum relapse has fallen from approximately 30% to 14% over recent decades – reflecting factors such as improved prepartum disease control, increased use of highly effective DMTs, and earlier reintroduction of DMT after delivery^1,2,12^ – incomplete recovery from relapses is associated with long-term disability accumulation.^13^ In addition, magnetic resonance imaging (MRI) studies have shown that women have greater T2 lesion volumes postpartum relative to pre-pregnancy, although few resumed DMT in the early postpartum period.^14,15^ Therefore, it is difficult to know whether the short-term biological processes associated with pregnancy are linked to long-term MS outcomes.

The impact of pregnancy after MS onset on long-term disease outcomes remains contentious.^16^ In MS, some studies have reported that pregnancy is associated with delayed diagnosis of clinically isolated syndrome,^17^ slower biological aging^18^ and reduced long-term disability, including a longer time to reach disability milestones^19,20^ and less disability accumulation over a 10-year period.^21^ However, other recent analyses have not found a beneficial effect.^22–25^ Differences in study outcomes may relate to study design and statistical approaches. A major criticism of past studies showing a benefit of pregnancy is the potential for indication bias.^24^ Women with milder disease may be more likely to have pregnancies and therefore appear to have better outcomes than their nulliparous peers.

An important question that requires further investigation is whether the timing of pregnancy influences its association with disability progression. MS-related inflammation is more pronounced at younger ages and shorter disease durations,^26^ and DMTs are most effective when initiated early in the disease course when inflammatory activity is greatest.^27,28^ Whether the immunomodulatory effects of pregnancy are also more pronounced during earlier disease stages, potentially leading to varying associations between pregnancy and disability outcomes at different points in the disease course, remains uncertain.

Our study aimed to examine the association between pregnancy and disability progression in a large cohort of women with mild relapse-onset MS, and whether this association differed by the timing of pregnancy.

## Methods

### Ethics approvals

This study was approved by the Alfred Health Human Research Ethics Committee and by institutional review boards at all participating centres. Written informed consent was obtained from all participants in accordance with the Declaration of Helsinki.

### Study design and data collection

This was a retrospective cohort study utilising longitudinal outcomes data from the MSBase Registry.^29^ MSBase is an international, prospective, observational MS clinical outcomes registry (World Health Organization International Clinical Trials Registry Platform registration ID: ACTRN12605000455662). Clinical assessments occur on average every 6 months. Data collected include participant demographics, disease phenotype, Expanded Disability Status Scale (EDSS) scores, relapse information, DMT use, and reproductive history.^30^ Data were extracted on 2 February 2022.

### Study population and inclusion criteria

Participants were recruited from 10 tertiary-referral MS specialist centres across Australia, the Czech Republic, and Kuwait that participate in the MSBase Registry. All participants completed detailed reproductive history surveys including history of miscarriages and induced terminations to supplement data recorded in MSBase.^29,30^ Inclusion criteria comprised women with relapse-onset MS aged between 18 and 45 years at MS diagnosis, with an EDSS score of 2.5 or below at baseline, and at least two additional EDSS scores recorded after baseline.

### Study endpoint

The study endpoint was defined as reaching a sustained EDSS score of 3 from a baseline EDSS score of 2.5 or less. This required achieving an EDSS score of 3 or higher and maintaining a score of 3 or higher on all (minimum 1) subsequent EDSS assessments, with a minimum of 6 months between assessments.

### Definitions

Baseline was defined as the time of the first EDSS score recorded in MSBase after MS onset.

Live births were defined as all pregnancies resulting in a live birth, whether term (>37 weeks gestation) or preterm (34–37 weeks gestation).

Pregnancy included all pregnancies, whether they ended in a termination, miscarriage, or live birth.

DMTs were grouped into three efficacy categories: low efficacy (interferon beta preparations (subcutaneous interferon beta-1a, interferon beta-1b, peginterferon beta-1a and intramuscular interferon beta-1a), glatiramer acetate, teriflunomide and azathioprine), moderate efficacy (dimethyl fumarate, fingolimod, siponimod, cladribine, daclizumab and cyclophosphamide) and high efficacy (natalizumab, ocrelizumab, alemtuzumab, rituximab, ofatumumab, mitoxantrone and stem cell transplant). Azathioprine and cyclophosphamide reflect off-label use recorded in MSBase.

### Statistical analysis

Data processing and statistical analyses were performed in R (version 4.2.2; R Foundation for Statistical Computing) or Stata (version 17; Stata Corp, College Station, TX, USA). Flexible parametric survival modelling was conducted with the *rstpm2* package in R and *stpm3* in Stata. Continuous variables were assessed for normality using the Shapiro–Wilk normality test and described as appropriate based on their distribution. All analyses were two-tailed.

### Flexible parametric survival models

To examine the influence of live-birth pregnancy on the time to sustained EDSS 3, we constructed several survival models based on flexible (Royston–Parmar) parametric regression.^31^ Time to this disability milestone was compared between women with a live-birth pregnancy after baseline (with or without pre-baseline pregnancies), and women with no live-birth pregnancies after baseline (including those with prior pregnancies and never-pregnant women). Live birth was modelled as a time-varying covariate (coded as 0 prior to first live birth and 1 thereafter). All models were adjusted for age at baseline, disease duration at baseline (including an interaction term between age and disease duration), baseline EDSS score, annualised relapse rate (ARR) in the 12 months prior to baseline, epoch of symptom onset, country of origin and high-efficacy DMT exposure modelled as a time-varying covariate.

Restricted cubic splines were applied where necessary to allow time-dependent effects for both the baseline and covariate log cumulative hazards. This approach permits time-varying coefficients (TVCs), enabling the hazard ratio (HR) for a covariate to change over time. TVCs were included for any covariate that violated the proportional hazards assumption, tested using Schoenfeld residuals, provided that model fit was improved. In our models, TVCs were specified for live birth, age at baseline, baseline EDSS and ARR in the 12 months pre-baseline.

Models with varying degrees of freedom on the baseline log cumulative hazard, and with and without TVC effects, were compared using the Akaike information criterion (AIC), and the best-fitting model was selected based on the lowest AIC. Our best-fitting primary model consisted of no interior knots (i.e. one degree of freedom) on the baseline log cumulative hazard, reducing it to a linear function that follows a standard Weibull distribution.

Model results are presented as standardised ‘effects’ – HRs, survival curves, restricted mean survival times (RMSTs) and their relevant contrasts where appropriate. Effects were computed under two counterfactual scenarios: one where every woman has never had a live birth and one where every woman has had one live birth. RMSTs at 5 and 10 years were estimated to assess absolute differences in disability-free survival between women with and without a live birth.^32^

### Subgroup analysis

In a subgroup analysis of women with a live birth after baseline, we examined the association between a relapse within 6 months postpartum and time to sustained EDSS 3. Only the first post-baseline live birth was included for each participant. The model was adjusted for the same covariates as the primary model, excluding live birth. Furthermore, RMSTs at 10 years were estimated to assess absolute differences in disability-free survival between women with and without a postpartum relapse.

### Sensitivity analyses

To assess the robustness of our results, we performed four sensitivity analyses. First, we filtered for women with a baseline within 12 months of MS symptom onset, reset baseline to symptom onset, and repeated the primary analysis. Second, we expanded the exposure to pregnancies of any duration, including live births, miscarriages and terminations. Third, we re-fitted the primary model with time to first live birth as an additional covariate to account for confounding by pregnancy timing. Finally, we restricted both groups to women with no pre-baseline pregnancies, comparing those with a live-birth pregnancy after baseline to never-pregnant women.

### Data availability

Sharing of de-identified patient-level clinical data may be possible in principle but requires permission from each contributing MSBase centre principal investigator (data controller). Requests will be reviewed and responded to within 4 weeks.

## Results

### Participant characteristics

We assessed the eligibility of 3965 women with MS from Australia, the Czech Republic and Kuwait who were enrolled in the MSBase Registry and had additionally completed a detailed reproductive history survey. A total of 2079 women with mild relapse-onset MS (EDSS score ⩽ 2.5 at baseline) met study inclusion criteria (Figure 1). Of these, 656 (31.5%) had at least one pregnancy after baseline (of whom 571 had at least one live birth), 805 (38.7%) had at least one pregnancy before baseline and none thereafter and 618 (29.7%) had never had a pregnancy. The women included in our primary analyses contributed a median of 6.8 (interquartile range (IQR): 4.0-10.0) person-years of follow-up, with a total of 15,178 person-years of follow-up for the cohort. Study participants had a median baseline EDSS score of 1.5 (IQR: 1.0–2.0). We assessed a median of 17 (IQR: 10.0–32.0) EDSS scores per participant. Cohort characteristics, including post-baseline MS DMT exposure, are reported in Table 1.

**Figure 1.**
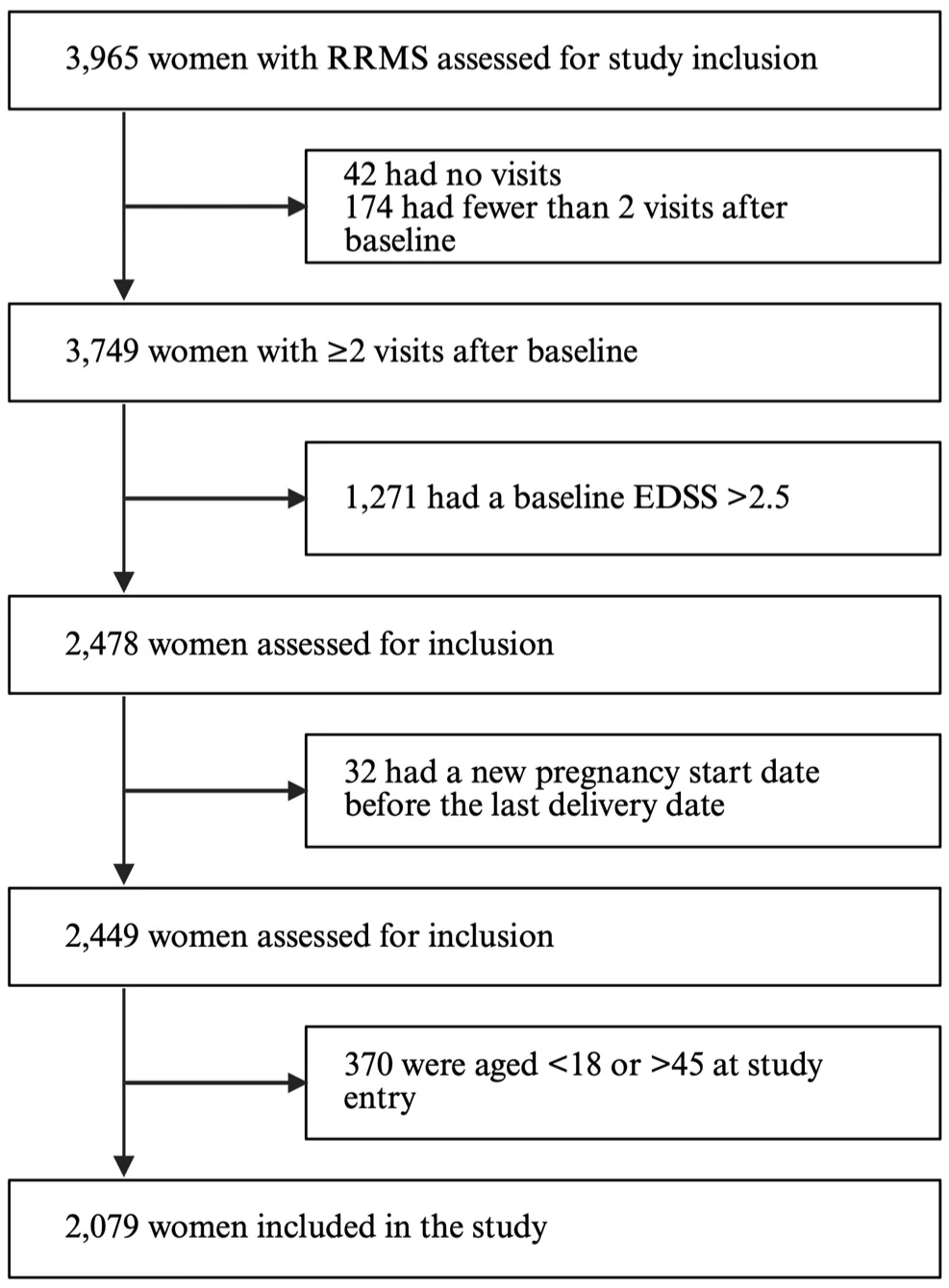
Flow diagram of study inclusion. Abbreviations: EDSS, Expanded Disability Status Scale; RRMS, relapsing-remitting multiple sclerosis.

**Table 1.** Participant demographic and baseline characteristics.

	Total (N = 2079)	At least one pregnancy after baseline (N = 656)	Pregnancy before baseline, none thereafter (N = 805)	Never had a pregnancy (N = 618)
Age, mean (SD), years	32.5 (7.1)	28.3 (4.6)	37.4 (5.7)	30.5 (7.2)
EDSS score, median (IQR)	1.5 (1.0–2.0)	1.5 (1.0–2.0)	1.5 (1.0–2.0)	1.5 (1.0–2.0)
Disease duration at baseline, median (IQR), years	1.7 (0.5–5.7)	1.6 (0.4–4.7)	3.1 (0.6–8.6)	1.1 (0.4–3.9)
Pregnancy prior to baseline, No. (%)	993 (47.8)	188 (28.7)	805 (100.0)	-
Year of first symptoms, No. (%)				
<1995	157 (7.6)	30 (4.6)	165 (13.2)	21 (3.4)
1995–2005	630 (30.3)	233 (35.5)	345 (32.5)	135 (21.8)
2005–2010	555 (26.7)	206 (31.4)	245 (23.2)	162 (26.2)
⩾2010	737 (35.4)	187 (28.5)	314 (31.1)	300 (48.5)
Post-baseline treatment exposure, No (%)				
Patients never on any DMT	191 (9.2)	56 (8.5)	89 (11.1)	46 (7.4)
Patients with a history of low-efficacy DMT	1,437 (69.1)	492 (75.0)	559 (69.4)	386 (62.5)
Patients with a history of moderate-efficacy DMT	763 (36.7)	254 (38.7)	270 (33.5)	239 (38.7)
Patients with a history of high-efficacy DMT	522 (25.1)	180 (27.4)	153 (19.0)	189 (30.6)
Post-baseline treatment exposure, median (IQR), years^a^				
Period with no treatment	0.0 (0.0–1.4)	0.9 (0.6–2.4)	0.0 (0.0–0.4)	0.0 (0.0–0.0)
Period on low-efficacy DMT	2.0 (0.0–5.5)	3.1 (0.0–6.7)	1.8 (0.0–5.3)	1.3 (0.0–4.6)
Period on moderate-efficacy DMT	0.0 (0.0–1.7)	0.0 (0.0–1.8)	0.0 (0.0–1.3)	0.0 (0.0–2.0)
Period on high-efficacy DMT	0.0 (0.0–0.0)^b^	0.0 (0.0–0.7)	0.0 (0.0–0.0)^b^	0.0 (0.0–1.7)
Follow-up duration, median (IQR), years	6.8 (4.0–10.0)	8.2 (5.3–11.1)	5.7 (3.2–9.3)	6.1 (3.5–9.6)

Abbreviations: DMT, disease-modifying therapy; EDSS, Expanded Disability Status Scale; IQR, interquartile range; SD, standard deviation.

Baseline was defined as the first EDSS visit after MS onset.

a Women with MS might have a history of multiple DMT commencements. The reported periods represent the cumulative duration of exposure to each DMT category across the entire follow-up.

b The range (minimum–maximum) of this variable in the total population and in women with a pregnancy before baseline and none thereafter was 0.0–17.5 and 0.0–15.3 years, respectively.

### Primary endpoint

In total, 522 women (25.1%) reached the study endpoint of sustained EDSS 3. By pregnancy history: 156 women (29.9%) who had at least one pregnancy after baseline reached sustained EDSS 3 over a median follow-up of 8.2 (IQR: 5.3–11.1) years per woman and 997 person-years; 275 women (52.7%) who had at least one pregnancy prior to baseline but none thereafter reached sustained EDSS 3 over a median follow-up of 5.7 (IQR: 3.2–9.3) years per woman and a total follow-up of 1232 person-years, and 91 women (17.4%) who had never been pregnant reached sustained EDSS 3 over a median follow-up of 6.1 (IQR: 3.5–9.6) years per person and 401 person-years.

### Association between live-birth pregnancy and disability progression

Time to sustained EDSS 3 was compared between 571 women who had at least one live-birth pregnancy after baseline and 1508 who did not have a live-birth pregnancy after baseline. Kaplan–Meier survival curves illustrating unadjusted group differences are shown in Figure 2. The primary flexible parametric survival model showed an association between live-birth pregnancy and longer time to sustained EDSS 3 for pregnancies occurring during the first 4.42 years of follow-up from baseline (Figure 3). Point-wise time-varying HRs for live-birth pregnancy are included in Table 2. For a live birth occurring 1 year after baseline, the HR was 0.48 (95% confidence interval (CI): 0.29, 0.82), indicating a 51% lower instantaneous hazard of reaching sustained EDSS 3 compared with women who had not had a live-birth pregnancy. This association diminished over the observation period, with the upper 95% CI crossing an HR of 1.0 at 4.42 years, and the HR associated with live birth remaining non-significant for the remainder of follow-up from baseline. The full results of the primary model are included in eTable 1 in the supplementary material.

**Figure 2.**
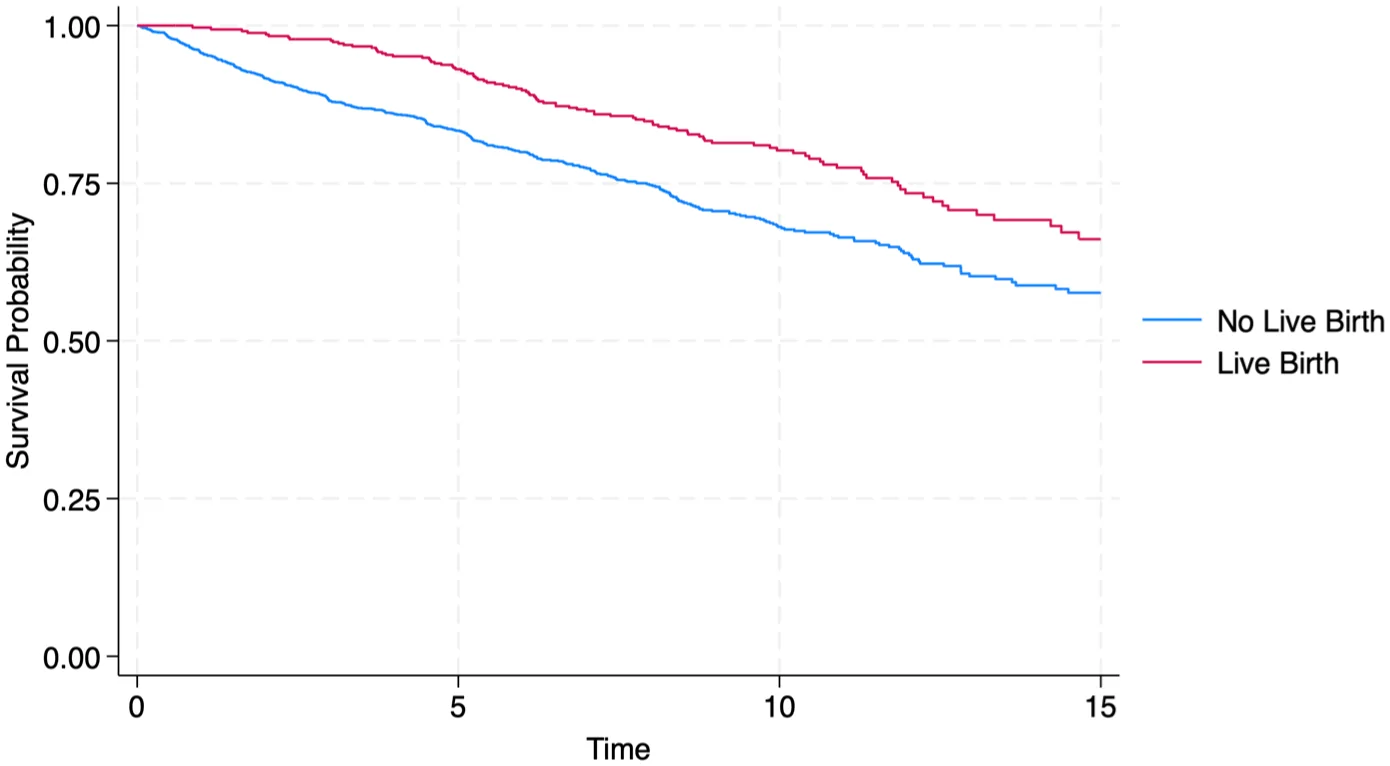
Kaplan–Meier survival curves for time to sustained EDSS 3 by pregnancy history. Abbreviation: EDSS, Expanded Disability Status Scale.

**Figure 3.**
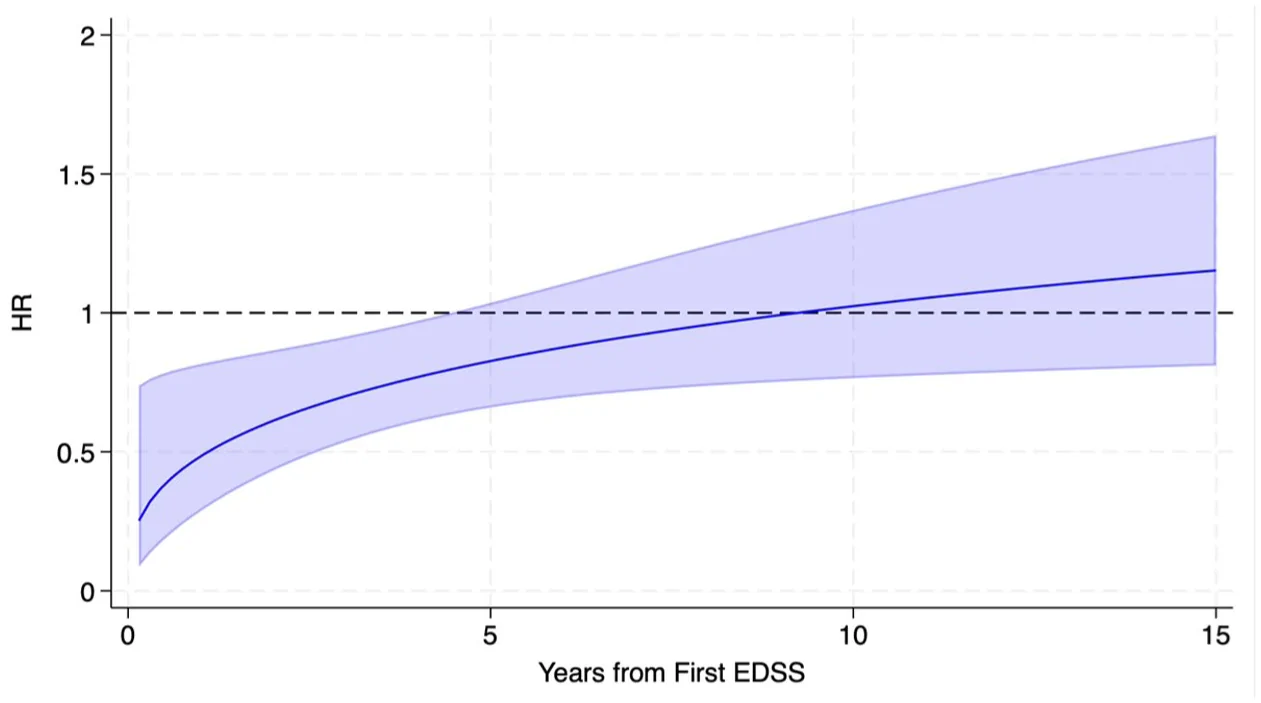
Time-varying hazard ratio for live-birth pregnancy on risk of sustained EDSS 3. Blue shaded areas represent 95% confidence intervals. Abbreviations: EDSS, Expanded Disability Status Scale; HR, hazard ratio.

**Table 2.** Point-wise time-varying HRs for the effect of live birth on risk of sustained EDSS 3.

Years after baseline	HR (95% CI)
1	0.48 (0.29, 0.82)
2	0.61 (0.43, 0.86)
3	0.70 (0.54, 0.91)
4	0.77 (0.61, 0.97)
4.25	0.78 (0.62, 0.99)
4.5	0.80 (0.64, 1.00)
4.75	0.81 (0.65, 1.02)
5	0.83 (0.66, 1.04)
10	1.02 (0.76, 1.37)
15	1.15 (0.81, 1.64)

Abbreviations: CI, confidence interval; EDSS, Expanded Disability Status Scale; HR, hazard ratio.

### Cumulative differences in disability-free survival

RMST analyses indicated a modest absolute difference in disability-free survival between women with and without a live-birth pregnancy, averaged across the follow-up period. At a 5-year horizon, women with a live birth had, on average, 0.20 years (~73 days) longer time to EDSS 3 compared to women without a live birth (95% CI: 0.10, 0.31). At 10 years, this difference increased to 0.51 years (~186 days) in favour of live birth (95% CI: 0.23, 0.79) (Figure 4).

**Figure 4.**
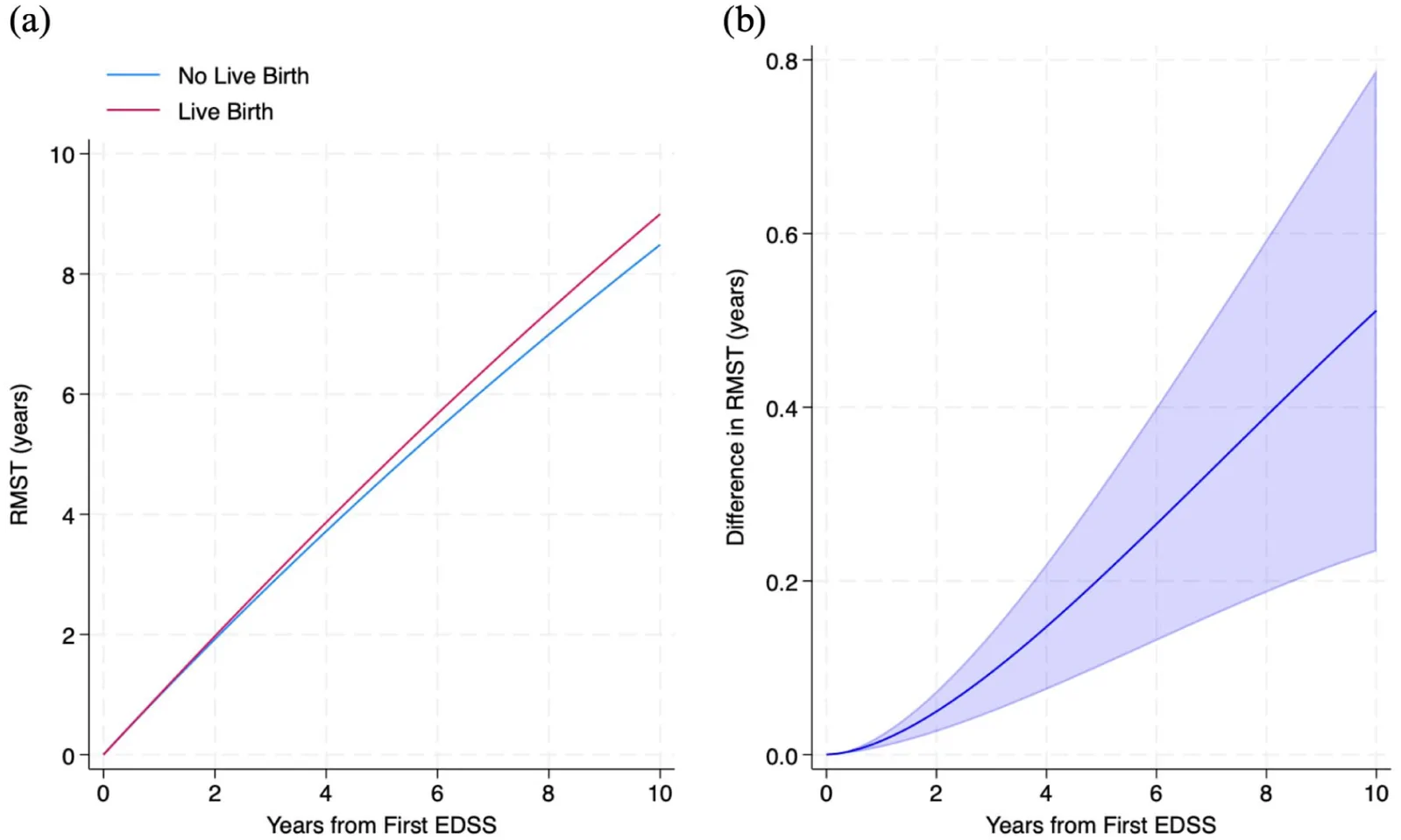
Cumulative effect of live-birth pregnancy on time to sustained EDSS 3: (a) RMST curves over 10 years from first EDSS assessment, stratified by live birth history. (b) Cumulative difference in RMST over a 10-year horizon, indicating the absolute delay in reaching sustained EDSS 3 associated with live-birth pregnancy. Blue shaded areas represent 95% confidence intervals. Abbreviations: EDSS, Expanded Disability Status Scale; RMST, restricted mean survival time.

### Association between postpartum relapse and disability progression

In the subgroup analysis of the 571 women with a first live-birth pregnancy, 139 (24.3%) had a relapse within 6 months postpartum. Experiencing a postpartum relapse was associated with a shorter time to sustained EDSS 3 compared to no postpartum relapse (HR: 1.61; 95% CI: 1.02, 2.55). The 10-year RMST difference was not statistically significant (−0.28 years, 95% CI: −0.57, 0.01).

### Sensitivity analyses

A sensitivity analysis in 833 women with baseline reset to MS symptom onset was consistent with the primary analysis, with a statistically significant HR of less than 1.0 observed during a portion of the early follow-up period for live-birth pregnancy (eFigures 1-2). Analyses including pregnancies of any duration mirrored the main findings (eFigures 3-4). Including time to first live birth as a covariate in the primary model also yielded similar results (eFigure 5). Finally, restricting both groups to women with no pre-baseline pregnancies (n = 1089) showed a pattern of association consistent with the main findings, with wider CIs reflecting the smaller sample size (eFigure 6).

## Discussion

Understanding the impact of pregnancy on long-term MS outcomes is important for family planning. However, the influence of pregnancy is difficult to ascertain due to many inherent biases when studying pregnancy impacts, particularly reverse causality. Using flexible parametric survival models, we sought to describe the association between pregnancy and disability outcomes in women with mild relapse-onset MS, and in particular whether this association differed by the timing of pregnancy during the follow-up period.

We chose to assess the association between pregnancy and time to a relatively early disability milestone, sustained EDSS 3. Past studies have often chosen moderate to severe disability endpoints when assessing pregnancy impacts, such as time to EDSS 4 or EDSS 6. Fortunately, the introduction of highly effective DMTs over recent decades has seen fewer people with MS reach these highly disabling milestones. Therefore, recent analyses of the impact of pregnancy on MS outcomes have, by necessity, censored most study participants for not reaching disability milestones of interest at the end of follow-up,^21,23,24^ making interpretation of the results difficult. Previous analyses also differ from ours in analytical framework and in their handling of key covariates such as DMT,^22,24^ underscoring the sensitivity of findings to methodological choices.

In our study of over 2000 deeply phenotyped women with relatively mild relapse-onset MS, live-birth pregnancy within 4.42 years of follow-up from baseline (first documented EDSS score) was associated with a longer time to sustained EDSS 3. The time-varying HR from the flexible parametric survival model indicated this association was strongest early in follow-up and attenuated over time, with no statistically significant difference between groups after 4.42 years. RMST analyses, by contrast, showed a modest absolute difference in cumulative disability-free survival favouring live birth of approximately 73 days at 5 years and 186 days at 10 years. Taken together, these analyses indicate that the association between earlier post-baseline live birth and slower disability accrual is concentrated in early follow-up, but accrues to a modest absolute difference over a 10-year horizon. To our knowledge, this is the first study to explore the time-varying nature of this association.

These results should be interpreted as describing broad temporal patterns rather than precise effects at any given time point, particularly at longer follow-up where data are more limited; the 4.42-year time should be regarded as a data-derived estimate from our model rather than a biologically defined or clinically actionable threshold. The slower disability accrual observed with pregnancies occurring earlier in follow-up may have several non-mutually exclusive explanations, such as a greater effect of pregnancy-induced immune adaptations during earlier disease stages when inflammatory activity is highest,^26^ and a healthy mother effect, whereby women with more stable disease may become pregnant earlier after baseline.

In the subgroup of women who had a live-birth pregnancy, those who experienced a relapse within 6 months postpartum reached sustained EDSS 3 more quickly than those who did not, although the absolute difference in cumulative disability-free survival at 10 years did not reach statistical significance. Postpartum relapse risk is influenced by multiple factors, including underlying disease activity, DMT interruption and the timing of resumption, and breastfeeding decisions.^1,25^ While incomplete recovery from a postpartum relapse may contribute to disability progression, this analysis indicates a prognostic association between postpartum relapse and disability accrual rather than establishing a direct causal relationship. These findings nonetheless support careful postpartum monitoring and treatment planning, consistent with current recommendations for peripartum MS care.^1,3,25^

## Limitations

Our study has several limitations. Causal interpretation relies on exchangeability (no unmeasured confounding), which is often unrealistic in observational studies. Residual confounding is therefore likely, and our findings should be interpreted as associations rather than causal effects. Several modelling considerations also warrant acknowledgement. Baseline was defined as the first EDSS visit, which does not always closely align with MS onset and cannot be directly translated into a clinically meaningful timeframe in relation to the disease course, although consistent results were observed in the sensitivity analysis using MS symptom onset as baseline. In addition, high-efficacy DMT was modelled as a single time-varying binary covariate; future studies are needed to examine more detailed aspects of peripartum DMT management. Finally, the cohort comprised women with mild relapse-onset MS who were enrolled in MSBase, retained in follow-up and completed a detailed reproductive survey. This cohort may not be representative of the wider MS population, and the findings cannot be extrapolated to women with moderate or severe disability or primary progressive MS.

## Conclusion

In our cohort study of women with mild relapse-onset MS, live-birth pregnancy occurring earlier in follow-up was associated with a longer time to disability progression, with the association attenuating over time but accruing to a modest cumulative difference at 10 years. Among women with a live-birth pregnancy, an early postpartum relapse was associated with faster disability progression, identifying postpartum relapse as a prognostic marker and supporting careful postpartum monitoring and treatment planning.

## Supplemental Material

sj-docx-1-msj-10.1177_13524585261464055 – Supplemental material for Association between pregnancy timing and disability outcomes in women with mild relapse-onset multiple sclerosis Supplemental material, sj-docx-1-msj-10.1177_13524585261464055 for Association between pregnancy timing and disability outcomes in women with mild relapse-onset multiple sclerosis by Jessica Shipley, Chao Zhu, Paul G Sanfilippo, Olga Skibina, Tomas Kalincik, Raed Alroughani, Bruce V Taylor, Richard Macdonell, Vicki E Maltby, Jeannette Lechner-Scott, Heidi N Beadnall, Anneke van der Walt, Helmut Butzkueven and Vilija G Jokubaitis in Multiple Sclerosis Journal

## Data Availability

Sharing of de-identified patient-level clinical data may be possible in principle, but requires permission from each contributing MSBase centre principal investigator (data controller). Requests will be reviewed and responded to within 4 weeks.
