# Supplementary material for: Association between pregnancy timing and disability outcomes in women with mild relapse-onset multiple sclerosis
Source: Mult Scler. 2026 Jul 29;32(12):1323–33. doi: 10.1177/13524585261464055 (PMC13672930; doi:10.1177/13524585261464055)
Supplement: Supplementary file 1 [file sj-docx-1-msj-10.1177_13524585261464055.docx]

**Supplementary material**

**Contents**

**eTable 1.** Results of the primary flexible parametric survival model

**eFigure 1.** Kaplan-Meier survival curves for time to sustained EDSS 3 for women seen from MS symptom onset

**eFigure 2.** Time-varying hazard ratio for live-birth pregnancy on risk of sustained EDSS 3 for individuals seen from MS symptom onset

**eFigure 3.** Kaplan-Meier survival curves for time to sustained EDSS 3 including pregnancies of any duration

**eFigure 4.** Time-varying hazard ratio for pregnancy of any duration on risk of sustained EDSS 3

**eFigure 5.** Time-varying hazard ratio for live-birth pregnancy on risk of sustained EDSS 3 including time to first live birth as a covariate

**eFigure 6.** Time-varying hazard ratio for live-birth pregnancy on risk of sustained EDSS 3, comparing women whose first live-birth pregnancy occurred after baseline to never-pregnant women

**eTable 1.** Results of the primary flexible parametric survival model

| **Variable** | **HR** | **95% CI** | **P value** |
| --- | --- | --- | --- |
| **Fixed effects** |  |  |  |
| Live birth | 0.33 | 0.15–0.72 | 0.01 |
| Age at baseline | 1.04 | 1.01–1.07 | 0.02 |
| Disease duration at baseline | 1.24 | 1.12–1.38 | <0.001 |
| Age x disease duration | 0.99 | 0.99–1.00 | <0.001 |
| Baseline EDSS score | 4.94 | 3.45–7.07 | <0.001 |
| High-efficacy DMT exposure^a^ | 0.82 | 0.60–1.13 | 0.22 |
| Annualised relapse rate | 0.72 | 0.65–0.80 | <0.001 |
| Epoch of symptom onset |  |  |  |
| 1995-2004 | 1.05 | 0.78–1.42 | 0.74 |
| 2005-2009 | 0.72 | 0.49–1.05 | 0.09 |
| ≥2010 | 0.45 | 0.28–0.72 | 0.001 |
| Country |  |  |  |
| Australia | 1.29 | 1.04–1.60 | 0.02 |
| Kuwait | 0.68 | 0.34–1.35 | 0.27 |
| **Time-varying coefficients** |  |  |  |
| Cumulative baseline hazard | 1.32 | 0.80–1.84 | <0.001 |
| Live birth | 0.34 | 0.04–0.65 | 0.03 |
| Age at baseline | 0.00 | -0.01–0.01 | 0.95 |
| Baseline EDSS score | -0.27 | -0.41– -0.12 | <0.001 |
| Annualised relapse rate | 0.11 | 0.08–0.15 | <0.001 |

^a^ Modelled as a time-varying covariate.

**Abbreviations:** CI, confidence interval; DMT, disease-modifying therapy; EDSS, Expanded Disability Status Scale; HR, hazard ratio.

**eFigure 1.** Kaplan-Meier survival curves for time to sustained EDSS 3 for women seen from MS symptom onset

**Abbreviations:** EDSS, Expanded Disability Status Scale; MS, multiple sclerosis.

**eFigure 2.** Time-varying hazard ratio for live-birth pregnancy on risk of sustained EDSS 3 for individuals seen from MS symptom onset

Blue shaded areas represent 95% confidence intervals.

**Abbreviations:** EDSS, Expanded Disability Status Scale; HR, hazard ratio; MS, multiple sclerosis.

**eFigure 3.** Kaplan-Meier survival curves for time to sustained EDSS 3 including pregnancies of any duration

**Abbreviations:** EDSS, Expanded Disability Status Scale.

**eFigure 4.** Time-varying hazard ratio for pregnancy of any duration on risk of sustained EDSS 3

Blue shaded areas represent 95% confidence intervals.

**Abbreviations:** EDSS, Expanded Disability Status Scale; HR, hazard ratio.

**eFigure 5.** Time-varying hazard ratio for live-birth pregnancy on risk of sustained EDSS 3 including time to first live birth as a covariate

Blue shaded areas represent 95% confidence intervals.

**Abbreviations:** EDSS, Expanded Disability Status Scale; HR, hazard ratio.

**eFigure 6.** Time-varying hazard ratio for live-birth pregnancy on risk of sustained EDSS 3, comparing women whose first live-birth pregnancy occurred after baseline to never-pregnant women

Blue shaded areas represent 95% confidence intervals.

**Abbreviations:** EDSS, Expanded Disability Status Scale; HR, hazard ratio.
